# Board-level AI literacy as a missing governance capability: a pilot study of boards and executives

**DOI:** 10.3389/frai.2026.1872783

**Published:** 2026-07-27

**Authors:** Darko Tipurić, Domagoj Hruška, Ivana Kovač

**Affiliations:** Faculty of Economics and Business, University of Zagreb, Zagreb, Croatia

**Keywords:** AI governance, board AI literacy, board competence, corporate sustainability, ESG reporting, human oversight

## Abstract

Corporate boards increasingly bear formal fiduciary responsibility for overseeing AI-driven strategic and sustainability decisions, yet their practical capacity to exercise such oversight substantively remains poorly documented. This study investigates whether a measurable AI governance capability gap exists between board-level and executive-level AI competence and examines the implications of this gap for the integrity of AI-driven ESG reporting. A pilot study (*n* = 26) of board members and senior executives documents a statistically significant governance capability asymmetry. Board AI literacy (BAL: M = 2.50, SD = 1.29) is significantly lower than executive AI competence (EAC: M = 3.70, SD = 0.95; Mann–Whitney *U* = 32.5, *p* = 0.026, Cohen’s *d* = 1.06). Board-level AI auditing capacity (M = 1.79) and ethical AI oversight (M = 1.86) approach the measurement floor, indicating that functional human oversight mechanisms are largely absent at governance level. Executive AI competence is strongly associated with organizational AI strategic orientation (*ρ* = 0.85, *p* = 0.002), consistent with a pattern in which AI-driven strategic transformation advances without commensurate board scrutiny. The findings suggest that boards may formally retain fiduciary responsibility for AI oversight while lacking the technical capability required to evaluate AI-generated strategic and sustainability outputs in an informed manner. In AI-driven ESG contexts, this capability deficit may increase the risk that sustainability disclosures are governed through formal endorsement rather than technically informed review. The study identifies board AI literacy as a missing governance capability in current corporate AI practice and offers preliminary empirical signals warranting larger-scale investigation of board-level AI oversight capacity.

## 1 Introduction

Corporate boards now routinely approve strategic decisions generated or substantially shaped by AI systems. They endorse sustainability disclosures produced by algorithmic ESG tools and receive management briefings synthesized by AI platforms whose methodological assumptions are rarely transparent at governance level. Whether boards possess the technical literacy to scrutinize—rather than simply ratify—these AI-generated outputs is a governance question with significant practical consequences that remains poorly documented.

The question is particularly consequential for corporate environmental sustainability. ESG reporting has undergone rapid AI integration: algorithmic tools quantify carbon footprints and supply chain exposure; AI-driven rating platforms aggregate outputs into investability scores directing capital allocation at global scale. Yet the alignment between AI-generated ESG disclosures and verified environmental performance is contested. Greenwashing—the systematic decoupling of ESG disclosure quality from environmental substance—has been identified as a structural feature of contemporary sustainability governance (Yu et al., 2020; Marquis et al., 2016; Berg et al., 2022). When boards lack the AI competence to evaluate the assumptions underlying the tools they formally oversee, that structural risk is compounded.

The board governance literature has extensively examined how board characteristics—diversity, independence, expertise, committee structure—predict environmental performance (García-Martín and Herrero, 2020; Yu et al., 2020). What it has not yet systematically measured is whether boards possess the AI governance capability necessary to exercise meaningful oversight of the algorithmic systems now embedded in sustainability decision-making. International AI governance frameworks—from the OECD AI Principles (OECD, 2023) to the EU Artificial Intelligence Act (European Parliament and Council, 2024)—explicitly mandate human oversight of high-stakes AI systems. Corporate sustainability governance is unambiguously such a domain. Yet board capacity to meet this oversight requirement has not been empirically examined.

This paper addresses two research questions. First, is a measurable AI governance capability gap observable at the board level, and does its configuration match theoretically predicted patterns? Second, what are the implications of this gap for the integrity of AI-driven ESG disclosure and for operationalizing human oversight of AI in corporate sustainability governance? To address the first question, we present findings from a pilot study (*n* = 26) of corporate board members and senior executives documenting a statistically significant, large-effect gap between board AI literacy and executive AI competence, near-floor scores on AI auditing and ethical oversight, and strong executive-level coupling between AI competence and organizational AI strategic direction. To address the second, we develop a theoretical interpretation drawing on institutional board competence suppression dynamics and algorithmic capture as institutional lenses. Given the pilot character of the study, the empirical findings are interpreted as indicative capability signals rather than population-level prevalence estimates.

This study makes three contributions to the emerging literature on corporate AI governance. First, it identifies board AI literacy as a distinct and currently undermeasured governance capability that conditions the substantive feasibility of human oversight of AI in strategic and sustainability decision domains. Second, it provides pilot empirical evidence of a measurable governance capability asymmetry between boards and executive management, documenting not only a board–executive AI competence gap but also critically low board-level AI auditing and ethical oversight capacity. Third, it advances preliminary practical implications for AI governance design by showing that formal governance structures alone may be insufficient where board-level AI capability remains underdeveloped. In doing so, the study extends existing board governance research beyond structural composition variables toward the measurement of governance competence in AI-integrated organizational environments.

The paper proceeds as follows. Section 2 reviews the board governance and AI oversight literature. Section 3 develops the theoretical framework. Section 4 presents research design and methodology. Section 5 reports results. Section 6 discusses findings and implications. Section 7 presents conclusions and practical governance implications.

## 2 Board governance capabilities and the AI oversight gap

### 2.1 Board characteristics and environmental governance: established findings and their limits

The empirical literature on board characteristics and environmental performance has generated genuine progress: board diversity, independence, and director expertise are consistently associated with improved ESG governance and reduced greenwashing (García-Martín and Herrero, 2020; Yu et al., 2020; Taglialatela et al., 2024), while sustainability governance structures—CSR committees, ESG-linked compensation, environmental reporting systems—are positively associated with environmental outcomes (Liu, 2024; Endrikat et al., 2020).

However, these associations are substantially weaker and more heterogeneous than the policy confidence in governance reform would suggest (Endrikat et al., 2020; Zubeltzu-Jaka et al., 2020), and the gap between formal governance adequacy and substantive environmental performance remains a persistent empirical pattern (Yu et al., 2020; Marquis et al., 2016). Established explanations—institutional isomorphism (DiMaggio and Powell, 1983; Bromley and Powell, 2012), legitimacy theory (Dowling and Pfeffer, 1975), and agency theory (Jensen and Meckling, 1976)—capture genuine dimensions of this decoupling but treat governance failure as a problem of formal configuration rather than institutional competence, and do not account for the specific epistemic conditions that AI-integrated governance demands.

### 2.2 AI governance and board digital competence

As AI systems become embedded in corporate governance processes, a distinct capability dimension emerges: board AI literacy. Director technological knowledge shapes both the strategic decisions boards approve and the quality of oversight they exercise (Chen and Hao, 2022; Fenwick et al., 2019). In an AI-integrated context, the relevant capability is the technical literacy required to evaluate AI system outputs, identify methodological limitations in AI-generated analytics, and interrogate assumptions embedded in AI-driven recommendations.

International governance frameworks have articulated this oversight requirement explicitly: the OECD AI Principles (OECD, 2023) and the EU AI Act (European Parliament and Council, 2024) establish human oversight as a core accountability requirement for AI systems in high-stakes domains, requiring that responsible actors possess the technical competence to meaningfully review AI-generated outputs (Jobin et al., 2019). In the ESG domain, the capability most at risk is the ability to evaluate whether algorithmic sustainability metrics accurately reflect environmental performance, or represent disclosure outputs optimized for rating criteria rather than environmental substance (Berg et al., 2022). Empirically documenting this capability gap across governance tiers is the primary empirical contribution of the present study.

### 2.3 The explanatory gap

The board-characteristics literature has mapped structural preconditions for effective environmental governance with considerable precision, but has underinvestigated whether boards possess the AI literacy necessary to scrutinize the algorithmic recommendations increasingly shaping strategic environmental decisions. This gap—between formal board composition governance and AI governance capability—motivates the theoretical framework in Section 3 and the empirical investigation in Sections 4–5.

## 3 Theoretical framework: governance capability deficits and institutional dynamics

Two analytical concepts anchor the interpretive framework. The first describes how institutional selection processes can systematically constrain the epistemic quality of governance actors. The second describes how AI systems can erode the independent judgment those actors are expected to exercise. Their interaction produces the governance capability configuration this study sets out to measure.

### 3.1 Morocracy: patronal selection and board competence suppression

Morocracy, introduced by Tipurić (2025), derives from the Greek *moros* (foolish) and *kratia* (to rule). The concept designates governance systems in which the systematic promotion of incompetence through loyalty-based appointment is a self-reproducing institutional feature rather than a deviation from meritocratic norms—sustained through patronal selection that privileges loyalty over competence, defensive management that prizes conformity over initiative, and a formal simulation of merit-based governance that systematically subverts it in practice (Tipurić, 2025).

For the purposes of this study, morocracy—among other possible institutional interpretations—functions as a lens for interpreting a specific observable pattern: boards that exhibit low AI governance capability while simultaneously acknowledging its importance. Where patronal selection logic governs board appointments, competence acquisition in domains that would enable critical interrogation of management strategy may be institutionally counterproductive. This interpretation is offered as a theoretical proposition warranting investigation in larger, longitudinal research designs; the pilot data are interpreted as preliminary evidence of a pattern consistent with it.

### 3.2 Algorithmic capture: AI integration and governance judgment

Algorithmic capture designates the progressive delegation of strategic and fiduciary judgment to AI-driven systems in ways that reduce rather than support substantive human oversight. AI systems are neutral only with respect to the variables their training data contains and the objectives their optimization functions specify—values that resist quantification, including much of what makes environmental sustainability consequential, are constitutively absent from their processing logic (Muller, 2018; O’Neil, 2016; Pasquale, 2020). In the ESG context, AI-driven rating systems trained on historical disclosure data may reward organizations that excel at producing high-ESG-score signals without corresponding environmental improvement (Yu et al., 2020; Chen and Hao, 2022). When boards rely on AI-generated ESG analytics without the technical literacy to interrogate their assumptions, they risk ratifying outputs optimized for algorithmic recognition rather than environmental accountability—a risk most acute precisely where board-level AI oversight capacity is lowest.

### 3.3 The governance capability gap as a compound condition

The two mechanisms introduced above do not operate in parallel isolation; when simultaneously present, they generate a compound institutional condition whose governance consequences exceed what either mechanism would produce alone. Patronal selection dynamics—of which morocracy represents one analytically sharp formulation—constrain the epistemic quality of the governance tier from the supply side. Where board appointment processes are governed primarily by relational proximity, political reliability, or organizational conformity rather than domain competence, the director pool is systematically filtered against technical expertise. Crucially, this filtering is not experienced as a deficit from within the logic of such systems: loyalty-based appointment reproduces itself precisely because competence in domains enabling the critical interrogation of management strategy—including, increasingly, AI systems—may be institutionally counterproductive to those who control appointment processes. The result is not a correctable individual knowledge gap but a structurally reproduced epistemic condition at the governance tier: the actors formally responsible for oversight consistently lack the capability that meaningful oversight would require.

Algorithmic capture compounds this supply-side constraint through a parallel demand-side mechanism. As AI systems become embedded in corporate strategy, operations, and sustainability reporting, technical AI competence becomes an increasingly consequential form of organizational knowledge—one that accumulates primarily at the management tier. This concentration is not necessarily the product of deliberate exclusion; it reflects the organizational reality that executives are the actors most directly and continuously engaged with AI systems in practice. The informational asymmetry that results is further amplified by reporting conventions: executive-to-board AI communication is typically delivered at summary level, stripping away the methodological assumptions, training data provenance, and optimization objectives that would need to be transparent for substantive board scrutiny to be exercised. A board without sufficient AI literacy to recognize what information is absent cannot effectively demand it; an executive tier operating without technically capable board oversight has limited structural incentive to provide it. Each mechanism thus reinforces the other: institutionally suppressed board competence reduces the pressure for informational transparency, while reduced informational transparency further constrains opportunities for board competence to develop.

The compound condition these mechanisms produce is what this study terms the board AI governance capability gap: a structural configuration in which the actor with formal oversight authority lacks substantive AI capability, while the actor with AI capability is not subject to informed independent review. In the ESG domain, where AI tools are increasingly used to generate the sustainability metrics and environmental disclosures that boards formally endorse, this misalignment carries direct accountability implications. AI-driven rating systems trained on historical disclosure data may reward signal optimization over genuine environmental performance (Yu et al., 2020; Berg et al., 2022); a board that cannot interrogate the generating systems cannot distinguish disclosure quality from environmental reality. Crucially, the gap is not unidimensional—a simple difference in AI knowledge between governance tiers. It is structural and multi-layered: it encompasses auditing capacity, ethical oversight capability, AI risk assessment, strategic engagement with AI as a governance agenda item, and the quality and frequency of the informational channels connecting executive and board. International AI governance frameworks—from the OECD AI Principles (OECD, 2023) to the EU AI Act (European Parliament and Council, 2024)—establish substantive human oversight as a core accountability requirement precisely because AI systems operating without technically informed review in high-stakes domains generate accountability failures that are difficult to detect and resistant to post-hoc correction (Jobin et al., 2019). The compound institutional condition identified here renders that requirement structurally difficult to fulfill.

**Figure 1:**
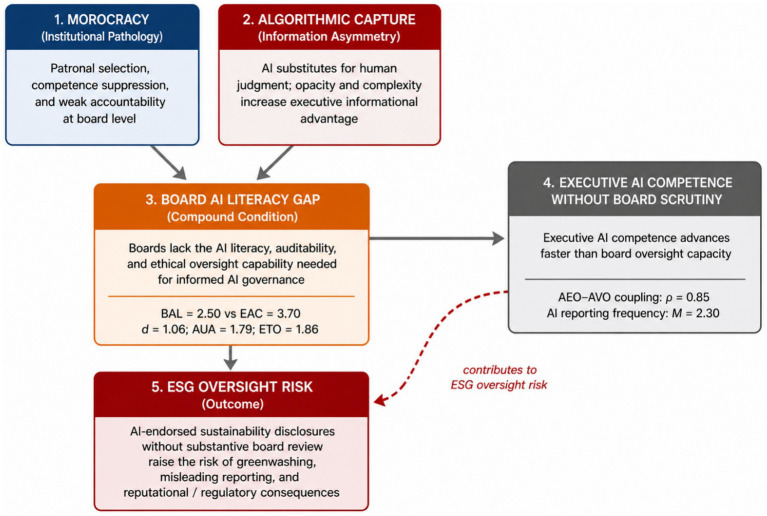
Theoretical model of the board AI governance capability gap. Institutional antecedents and algorithmic capture converge to produce a board-level AI governance capability gap, reinforced by executive–board informational asymmetry. The downstream outcome is ESG oversight risk in AI-mediated sustainability disclosure.

Figure 1 summarizes the theoretical architecture developed across Sections 3.1 through 3.3. The model represents two institutional antecedents—patronal selection dynamics and algorithmic capture—as converging inputs that produce a board-level AI governance capability gap, operationally expressed in measurably low board AI literacy, near-absent AI auditing and ethical oversight capacity, and weak board engagement with AI governance as a strategic function. An executive–board informational asymmetry, reinforced by the tight coupling between executive AI competence and organizational AI strategic direction, appears in the model as a mechanism that deepens the gap independently of the antecedent conditions. The downstream governance outcome—ESG oversight risk—represents the domain in which the capability gap has its most consequential practical expression, given the AI-integrated character of contemporary sustainability disclosure. This theoretical architecture generates a set of empirically tractable observable implications, which the pilot study reported in Sections 4 and 5 evaluates with a purposive sample of corporate board members and senior executives.

## 4 Research design and methodology

### 4.1 Study design

The study employs a mixed-methods design integrating a theoretical framework with a cross-sectional pilot survey. The empirical component tests whether the governance capability logic advanced in Section 3 generates observable symptom patterns—specifically whether a measurable asymmetry exists between board AI literacy and executive AI competence, and whether AI oversight structures show correspondingly low scores. The pilot study is positioned as preliminary evidence of the predicted pattern rather than confirmatory hypothesis testing; the sample size is insufficient for the latter, and the study is designed as a foundation for a larger-scale research program.

### 4.2 Sample and data collection

The sample consists of 26 governance professionals recruited through purposive sampling in Croatia, required to have current or recent experience as board members, supervisory board members, management board executives, or senior governance advisors. Data were collected via structured self-administered online questionnaire in two respondent-specific sections: board and supervisory board members completed Section B (AI governance at board level, 20 Likert items), and management executives completed Section C (AI governance at executive level, 20 Likert items), yielding subsamples of *n*₂ = 14 (board tier) and *n*₃ = 10 (executive tier). Table 1 presents the sample characteristics.

**Table 1:** Sample characteristics (*n* = 26).

Characteristic	Category	*n*	%
Position	Supervisory board member	10	38.5
	Management board member	9	34.6
	Board of directors member	3	11.5
	Advisor/board collaborator	4	15.4
Gender	Male	20	76.9
	Female	6	23.1
Age	31–40	6	23.1
	41–50	14	53.8
	51–60	4	15.4
	61–70	2	7.7
Sector	Energy and infrastructure	15	57.7
	Industry and manufacturing	4	15.4
	Other sectors	7	26.9
Organization size	1,000+ employees	14	53.8
	50–249 employees	7	26.9
	Fewer than 49 employees	4	15.4
	250–999 employees	1	3.8
Engagement recency	Currently active or within last year	20	76.9
	2–5 years ago	4	15.4
	More than 5 years ago	2	7.7

### 4.3 Survey instrument and variable operationalization

The survey instrument was developed in Croatian and comprises five thematic clusters within each section: AI strategic orientation, AI literacy and competence, AI in decision-making, AI risk governance, and AI organizational culture. All items use a five-point Likert scale (1 = strongly disagree to 5 = strongly agree).

Five variables are central to the analysis. Conceptually, board AI literacy is defined in this study as the collective capacity of a board to understand, interrogate, and critically evaluate the outputs, assumptions, and limitations of AI systems used in governance relevant domains, to a degree sufficient for informed oversight rather than formal ratification. Executive AI competence is the parallel capacity at the management tier, oriented toward the deployment and operational use of AI in the executive role. These definitions guided item selection. Board AI literacy (BAL) is operationalized as item B2.4, “The overall level of AI literacy in this board is sufficient for informed strategic decision-making.” Executive AI competence (EAC) is operationalized as item C4.3, “I consider my personal AI competencies adequate for the requirements of my managerial role.” The Governance Gap is defined as the difference between EAC and BAL means. AI auditing capacity (AUA) is item B4.3, “The board regularly conducts or oversees independent audits of AI systems used in the organization,” and ethical oversight of AI (ETO) is item B4.2, “The board actively monitors the ethical application of AI, including issues of algorithmic bias and data protection.” Organizational AI vision orientation (AVO) is the composite mean of the AI strategy section (C1.1–C1.4); executive AI competence orientation (ACO) is the composite mean of the AI competence and culture section (C4.1–C4.4).

An important methodological limitation must be stated here: both focal comparative variables, BAL and EAC, are operationalized as single Likert items. This is a known limitation of pilot organizational surveys operating under sample-size and instrument-length constraints. Single-item operationalizations have lower construct validity and reliability than validated multi-item scales, and the central finding of this study—the Governance Gap—rests on this single-item comparison. Readers should treat the reported means, gap magnitude, and effect size as preliminary estimates requiring replication with validated multi-item instruments before being interpreted as robust measures of board AI literacy and executive AI competence. The decision to retain these single focal indicators was guided by pilot stage questionnaire economy and respondent accessibility considerations. The authors fully acknowledge that the present findings should be interpreted as exploratory measurements pending future scale validation. To support transparency and replication, we record the following. All focal items use the common five point Likert anchoring (1 = strongly disagree to 5 = strongly agree) reported above. The exact wording of each focal item (B2.4, C4.3, B4.3, B4.2) is given verbatim in this section. The two composite variables are formed as unweighted means of their constituent items, ACO from items C4.1 to C4.4 and AVO from items C1.1 to C1.4, all drawn from the five thematic clusters of the instrument described at the start of this section. The full instrument, including all items not used as focal indicators, is available from the corresponding author on request. Taken together, this reporting is intended to allow a replication study to reconstruct the focal measures exactly while substituting validated multi item scales for the single item indicators where appropriate.

### 4.4 Statistical analysis

Descriptive statistics are reported for all focal variables. The Governance Gap (EAC vs. BAL) is tested using the Mann–Whitney *U* test, preferred over parametric alternatives because both variables are measured at the ordinal level on bounded five-point Likert scales, the groups are independent with unequal and small cell sizes (*n*₁ = 14, *n*₂ = 10), and the non-parametric approach is more robust to violations of variance homogeneity. While Shapiro–Wilk tests did not indicate departures from normality (Board: *W* = 0.91, *p* = 0.14; Executive: *W* = 0.91, *p* = 0.29), the ordinal measurement level justifies the non-parametric choice independently. Effect size is reported as Cohen’s d from pooled standard deviation to facilitate comparability with the governance literature. The executive AI competence–vision coupling is assessed using Spearman’s rank correlation. All tests are two-tailed, *α* = 0.05. To assess the robustness of the central comparison under the small sample, the Governance Gap was examined with both the non parametric Mann–Whitney *U* test and a supporting inspection of group medians and distributional overlap. The two converge on the same conclusion of a pronounced between tier separation, and the effect remains large under each. Because the sample is small, these robustness observations are reported as supporting evidence for the direction and approximate magnitude of the gap rather than as precise stability estimates, consistent with the pilot framing stated in Section 4.5.

### 4.5 Methodological limitations of the pilot design

The sample is small, purposively recruited, cross-sectional, and geographically concentrated in Croatia with sectoral concentration in energy and infrastructure (57.7%). These features preclude statistical generalization. The study is designed to detect whether the predicted governance capability pattern is present, not to estimate its prevalence. All measures are self-reported, introducing the possibility of systematic overstatement of AI competence or understatement of governance deficits. The cross-sectional design precludes causal inference. These limitations are inherent to the pilot design and are addressed in Section 6. One scope limitation warrants explicit statement here. The environmental sustainability and ESG dimension, although central to the study’s theoretical framing, is not measured directly through any survey item. The instrument captures board and executive AI governance capability, not ESG disclosure quality or environmental outcomes. The ESG relevance of the findings is therefore developed at the level of governance preconditions rather than measured ESG performance. The study’s claim is that board AI literacy is a necessary capability condition for substantive oversight of AI generated ESG outputs, and that its near absence describes a governance setting in which such oversight cannot be exercised in an informed manner. This is an analytically grounded inference about a governance condition, not a direct empirical observation of ESG misreporting, and it is treated as such throughout.

## 5 Results

The results are organized around three interlocking findings. First, a statistically significant, large-effect gap exists between board AI literacy and executive AI competence (Section 5.2). Second, executive AI competence is strongly coupled with organizational AI strategic direction while information flow toward the board remains sparse (Section 5.3). Third, every operational mechanism through which boards would exercise AI oversight—auditing, risk assessment, ethical monitoring, strategic engagement—registers near the measurement floor (Section 5.4). Taken together, these three findings describe a coherent governance capability configuration in which formal oversight authority and substantive oversight capacity are systematically misaligned.

### 5.1 Descriptive statistics

All survey items were scored on a five-point Likert scale anchored at 1 (“strongly disagree”) and 5 (“strongly agree”). For items measuring governance capability—whether a board conducts AI audits, whether its AI literacy is adequate, whether it actively monitors algorithmic risk—scores below 3.0 indicate that respondents, on average, disagree with the statement; scores approaching 1.0–2.0 indicate near-uniform disagreement across respondents. This interpretive frame is important for reading Table 2: a mean of 1.79 on an AI auditing item does not indicate that auditing is merely uncommon—it indicates that the typical board respondent actively disputes that their board conducts meaningful AI audits at all.

**Table 2:** Descriptive statistics for focal variables.

Variable	Description	*n*	M	SD	Mdn
Board/supervisory board (*n* = 14)					
B2.4	AI literacy (focal: BAL)	14	2.50	1.29	2.50
B4.2	Ethical oversight of AI (ETO)	14	1.86	0.86	2.00
B4.3	AI auditing capacity (AUA)	14	1.79	0.80	2.00
B1.4	Board AI strategy engagement	14	2.00	0.96	2.00
B3.1	Board-level AI risk assessment	14	1.64	0.74	2.00
B5.3	Board awareness: AI requires future readiness	14	3.21	1.05	3.00
Executive management (*n* = 10)					
C4.3	AI self-competence (focal: EAC)	10	3.70	0.95	4.00
C4	AI competence composite (ACO; C4.1–C4.4)	10	3.35	0.88	3.25
C1	AI vision orientation composite (AVO; C1.1–C1.4)	10	3.18	0.74	3.25
C1.3	Frequency of AI reporting to board	10	2.30	1.06	2.00
C5.2	Quality of board–management AI communication	10	2.50	1.08	2.50

All items scored on a 1–5 Likert scale. BAL, board AI literacy (B2.4); EAC, executive AI competence (C4.3); AUA, AI auditing capacity (B4.3); ETO, ethical oversight of AI (B4.2); ACO, executive AI competence composite (C4); AVO, AI vision orientation composite (C1). M, mean; SD, standard deviation; Mdn, median.

**Figure 2:**
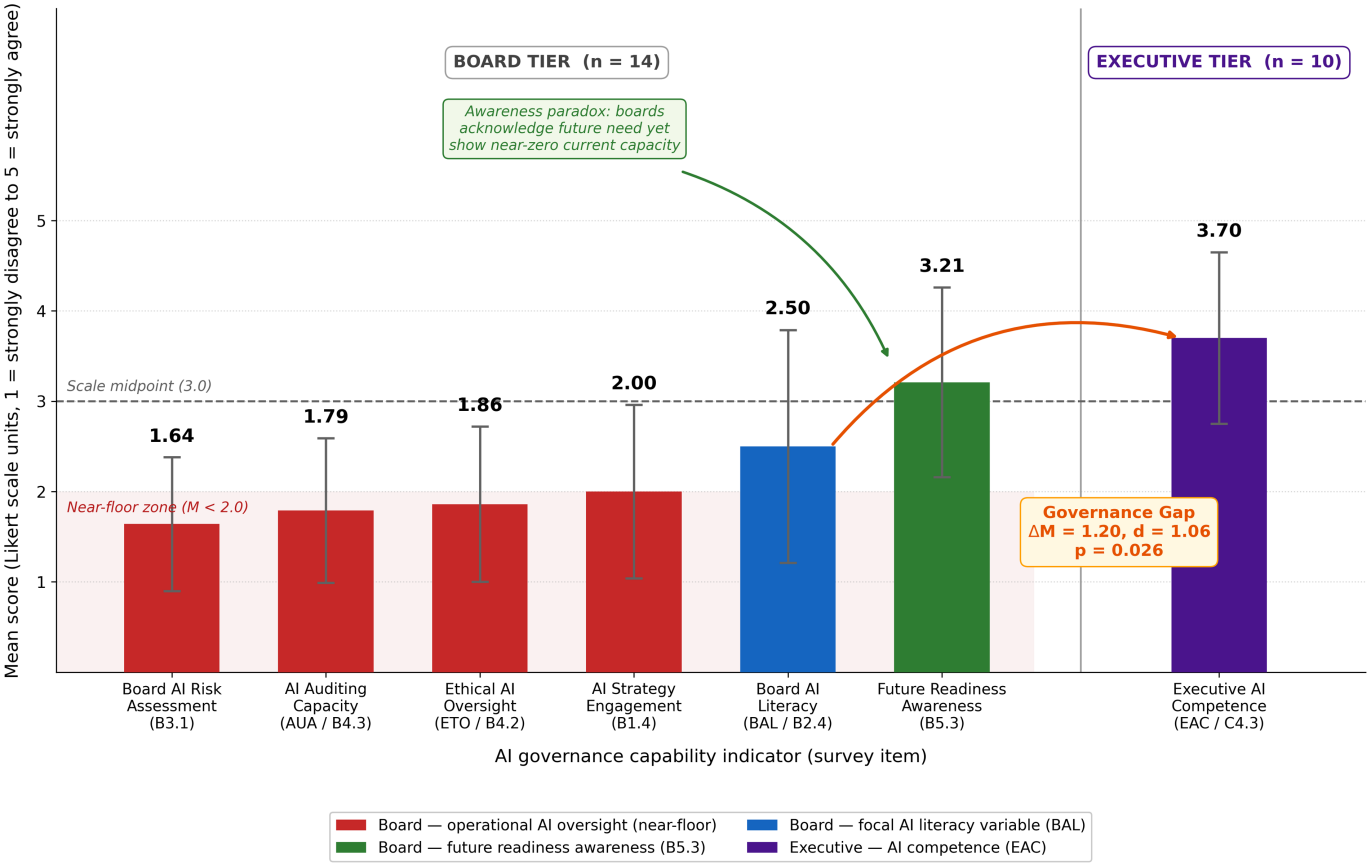
Comparative AI governance capability profile across board and executive tiers. Mean scores (±1 SD) on focal AI governance variables measured on a five-point Likert scale. Board-level indicators cluster in the low-capacity range, with auditing, ethical oversight, and AI risk assessment approaching the measurement floor, while executive AI competence is visibly higher. The highlighted bracket denotes the empirically observed governance gap between board AI literacy and executive AI competence (*d* = 1.06, *p* = 0.026).

Inspection of Table 2 reveals an interesting distributional pattern. All six board-tier indicators fall at or below the scale midpoint (M ≤ 2.50); five of the six fall below 2.50; three fall below 2.00. No board-tier operational AI governance variable—covering auditing, risk assessment, ethical oversight, strategy engagement, or overall AI literacy—reaches the neutral midpoint of the scale. By contrast, the executive-tier focal variable (EAC) registers M = 3.70, above the midpoint and approaching the “agree” region of the scale. Figure 2 renders this profile visually: the board-tier bars (left side) are distributed in the lower half of the chart, with three bars in the shaded near-floor zone, while the executive-tier bar (right side) stands apart at a qualitatively higher level.

### 5.2 The governance gap: board AI literacy versus executive AI competence

The study’s primary research question asks whether a measurable AI governance capability gap exists between board-level and executive-level AI competence. The descriptive data already suggest a pronounced asymmetry; the inferential analysis confirms it.

Board AI literacy (BAL), operationalized as item B2.4—*“The overall level of AI literacy in this board is sufficient for informed strategic decision-making”*—registered M = 2.50 (SD = 1.29, Mdn = 2.50) across the 14 board-tier respondents. A mean of 2.50 falls below the scale midpoint, indicating that the average board respondent tends to disagree that their board’s collective AI literacy meets the threshold for competent AI governance. The standard deviation of 1.29—broad relative to the scale range—indicates meaningful heterogeneity in board respondents’ assessments: some boards apparently experience higher AI literacy than others, even within this modest sample. Nevertheless, the central tendency is clearly below adequacy on the scale provided.

Executive AI competence (EAC), operationalized as item C4.3—*“I consider my personal AI competencies adequate for the requirements of my managerial role”*—registered M = 3.70 (SD = 0.95, Mdn = 4.00) across the 10 executive-tier respondents. A mean of 3.70 lies substantially above the midpoint, in the “agree” region of the scale, indicating that the typical executive respondent considers their AI competence adequate for their role. The lower standard deviation (0.95 vs. 1.29 for BAL) reflects greater homogeneity in executive self-assessments: executives tend to agree they are AI-competent.

The raw between-tier difference—1.20 scale points—represents a separation of nearly one-quarter of the total scale range. This descriptive gap is corroborated by the inferential test: the Mann–Whitney *U* test, appropriate for the ordinal measurement level and unequal group sizes of this design, yielded *U* = 32.5, *p* = 0.026 (two-tailed). The null hypothesis of distributional equality between board and executive AI competence scores is rejected at the conventional threshold. Effect size, estimated as Cohen’s d from pooled standard deviation, was *d* = 1.06. By convention (Cohen, 1988), values above 0.80 are classified as large. This magnitude indicates that the observed between-tier gap is not a marginal statistical artefact but a substantively pronounced asymmetry. This between-tier capability disparity, the Governance Gap, constitutes the study’s central empirical finding.

### 5.3 Executive AI competence and organizational AI vision orientation

The second set of findings concerns how AI capability at the executive tier relates to the organizations’ overall AI strategic direction—and what this relationship implies for board oversight when board AI literacy is simultaneously low. The analysis examines the correlation between two composite variables within the executive subsample (*n* = 10): the executive AI competence composite (ACO; items C4.1–C4.4) and the organizational AI vision orientation composite (AVO; items C1.1–C1.4).

Spearman’s rank correlation between ACO and AVO yielded *ρ* = 0.85 (*p* = 0.002). A correlation of this magnitude—*ρ*^2^ ≈ 0.72—indicates that approximately 72% of the variance in organizational AI vision orientation across the executive subsample is statistically associated with executive AI competence. In practical terms, this implies that how ambitiously and how concretely the organization deploys and plans AI is, to a very substantial degree, a function of how competent the executive leadership is in AI—not of board strategic direction alone. The executive tier is not merely implementing a vision that boards have shaped; it appears to be generating and propelling that vision largely from within its own competence base.

The governance consequence of this executive-driven AI integration becomes acute when the transmission channel between executive and board is considered. Executives report that they bring AI topics to the board infrequently: AI reporting frequency (C1.3) registered M = 2.30 (SD = 1.06, Mdn = 2.00), indicating that executive-to-board communication on AI strategy occurs below the “occasionally” threshold. Board–management communication quality on AI topics (C5.2) was rated M = 2.50 (SD = 1.08, Mdn = 2.50). Taken together, these indicators describe a structural configuration in which AI strategic capability concentrates at management tier, AI strategy is predominantly executive-driven, and yet the flow of AI-related information upward to the oversight tier is sparse. This is the institutional setting in which algorithmic capture—progressive executive dependence on AI-driven strategic outputs without independent board review—finds its most favorable structural conditions.

### 5.4 Critical deficits in AI auditing and ethical oversight

The third dimension concerns the specific operational mechanisms through which meaningful AI oversight would actually be exercised—auditing, risk assessment, ethical monitoring, and strategic engagement with AI as a governance agenda item.

AI auditing capacity (AUA, item B4.3: *“The board regularly conducts or oversees independent audits of AI systems”*) registered M = 1.79 (SD = 0.80, Mdn = 2.00). With a mean below 2.0, board respondents actively disagree that their boards engage in AI system audits—the median respondent falls squarely in the “disagree” category. Ethical oversight of AI (ETO, item B4.2) registered M = 1.86 (SD = 0.86, Mdn = 2.00), indicating consistent disagreement that boards exercise active ethical oversight of AI deployments. Board-level AI risk assessment (B3.1: M = 1.64, SD = 0.74, Mdn = 2.00) is the lowest mean in the entire dataset—the variable on which board respondents most strongly and most consistently dispute that the relevant governance function exists in their organization. Board AI strategy engagement (B1.4: M = 2.00) sits at the boundary of the near-floor zone. Across all four operational dimensions—auditing, ethical oversight, risk assessment, strategy engagement—not a single variable exceeds M = 2.00. This is not a pattern of uneven capacity, with some oversight mechanisms functional and others absent; it is near-uniform low capacity across every operational channel of board-level AI governance.

Figure 2 renders this pattern visually: the four leftmost bars (B3.1, AUA, ETO, B1.4) in the board-tier cluster all fall within or at the boundary of the shaded near-floor zone. Reading left to right within the board tier, one observes a stepped ascent from risk assessment (M = 1.64) through auditing and ethical oversight (M ≈ 1.80–1.86) to strategy engagement (M = 2.00) and overall AI literacy (BAL, M = 2.50)—all still well below the scale midpoint.

One board-level indicator breaks sharply from this pattern: board awareness that AI governance will require greater future preparedness (B5.3: M = 3.21, SD = 1.05, Mdn = 3.00). This is the highest board-tier mean in the study, exceeding the scale midpoint by 0.21 points and visible in Figure 2 as the bar rising clearly above the board-tier cluster. The juxtaposition is theoretically consequential: board members collectively acknowledge that AI governance is increasingly important and that greater preparedness will be required, while simultaneously reporting near-complete absence of current operational capacity. Under standard human capital logic—where awareness of a capability gap motivates corrective investment—this pattern would be transitional. The persistence of near-floor operational capacity alongside above-midpoint future-readiness awareness raises an alternative interpretation: that in this institutional context, the recognition of AI governance importance and the acquisition of AI governance capability are decoupled processes. Section 6 examines this interpretation through the theoretical lenses developed in Section 3.

Taken together, the three result sets describe an internally coherent governance capability configuration: board AI literacy is low; operational AI oversight mechanisms are near-absent across every measured dimension; executive AI competence is substantially higher and tightly coupled with organizational AI strategic direction; the upward information channel functions at low intensity; and board awareness of AI governance’s future importance coexists with near-zero current operational capacity. This pattern is consistent with the theoretical prediction of a compound governance capability gap in which the actor with formal oversight authority lacks the substantive capability to exercise it, and the actor with AI capability is not subject to effective board review.

## 6 Discussion

### 6.1 Principal findings: a board-level AI governance capability gap

The primary finding, a statistically significant and large effect Governance Gap (BAL: M = 2.50; EAC: M = 3.70; *d* = 1.06, *p* = 0.026), is the study’s central empirical contribution within the bounds of a pilot design. Given the small purposive sample, the reported effect magnitude is best read as an indicative signal of a pattern rather than a precise population estimate, and the interpretation that follows is advanced in that exploratory spirit. Several alternative interpretations of this gap are possible: a temporary transition as boards catch up with executive AI adoption; rational oversight delegation to specialist committees; or resource and time constraints particular to the sample.

The configuration of supplementary findings, however, constrains these interpretations. Near-floor scores on AI auditing (M = 1.79), ethical oversight (M = 1.86), risk assessment (M = 1.64), and strategic engagement (M = 2.00) indicate not partial capability but near-complete absence of operational AI governance mechanisms at board level across all measured dimensions. This breadth of deficit is not consistent with rational delegation (which would show concentrated capability in specific committee functions) or transitional adjustment (which would show recent upward movement). It is consistent with systematic, organization-wide absence of the epistemic preconditions for substantive AI oversight.

The motivated non-preparation pattern—simultaneous recognition of future AI governance importance (M = 3.21) and near-zero operational capacity—adds a theoretically important dimension. Under a human capital model, competence-gap awareness generates corrective investment. The persistence of this pattern is consistent with the morocratic institutional lens (Tipurić, 2025), which, among other possible institutional interpretations, predicts that where patronal selection governs board appointments, competence acquisition in domains enabling critical interrogation of management decisions may face institutional disincentives. We advance this interpretation as a preliminary empirical signal, not as confirmation of the theoretical proposition, pending replication in larger designs. Alternative explanations remain possible, including sector-specific digital maturity differences and generational technology exposure effects, which this pilot design cannot fully disentangle.

### 6.2 The executive–board coupling and AI governance implications

The *ρ* = 0.85 coupling between executive AI competence and organizational AI vision orientation (*p* = 0.002) indicates that AI-driven strategic transformation is predominantly executive-led, with over 70% of variance in organizational AI direction associated with executive AI competence. Combined with low board AI literacy and infrequent AI reporting to boards (C1.3: M = 2.30), this describes a governance configuration in which the actor with formal oversight authority lacks the capability, while the actor with AI capability is not subject to effective board oversight. This is precisely the organizational structure in which algorithmic capture finds its most favorable structural conditions: executive-driven AI integration advances with AI-generated recommendations accumulating strategic authority without commensurate independent board review—and where the OECD (2023) and EU AI Act (European Parliament and Council, 2024) human oversight requirements are operationally unmet.

### 6.3 Implications for AI-driven ESG disclosure

This study did not measure ESG disclosure quality or environmental performance directly, and the implications developed in this section concern governance preconditions rather than observed ESG outcomes. With that scope stated explicitly, the reasoning is as follows. In organizations where AI tools are used for ESG reporting, environmental monitoring, or supply chain assessment, board capacity to evaluate those tools is a necessary condition for substantive environmental accountability. Where that capacity is absent—as this study preliminarily documents—AI-driven ESG reporting risks decoupling from environmental governance in any substantive sense. Tools generate outputs that can be disclosed and rated, but no governance actor with sufficient technical competence is positioned to evaluate whether those outputs correspond to environmental reality.

The AI auditability dimension is particularly significant. Ethical AI principles—transparency, auditability, human oversight—are codified in international governance frameworks precisely because AI systems operating without adequate human oversight in high-stakes domains generate accountability failures resistant to post-hoc correction (Jobin et al., 2019). When boards endorsing AI-generated ESG disclosures cannot evaluate the assumptions or methodological choices of the generating systems, the human-in-the-loop requirement is operationally absent. The near-floor AI auditing score (M = 1.79) is the governance-level correlate of this structural risk. The present study therefore does not claim direct measurement of ESG misreporting. It identifies a governance capability condition under which such risks may become structurally more likely, and the strength of this claim is bounded accordingly. The link between the measured capability deficit and any actual ESG disclosure failure is theoretical, and establishing it empirically requires linkage studies of the kind set out in Section 6.4.

### 6.4 Limitations and future research

The sample (*n* = 26; *n*₁ = 14 board, *n*₂ = 10 executive) is small, cross-sectional, purposively recruited, and geographically concentrated in Croatia with sectoral concentration in energy and infrastructure. These features preclude prevalence claims. Effect sizes in small samples carry substantial estimation variance: the large Cohen’s *d* = 1.06 and *ρ* = 0.85 should be treated as pilot-stage signals requiring replication rather than stable population parameters. Self-report bias cannot be excluded.

The single-item operationalization of both central variables (BAL: B2.4; EAC: C4.3) is the most consequential methodological limitation of this study. As noted in Section 4.3, the central Governance Gap finding rests on a single-item comparison in each group. While this is an accepted approach in pilot research with instrument-length constraints, it limits the precision and construct validity of the reported gap. This limitation requires explicit resolution in follow-up research through validated multi-item AI literacy and AI competence scales. The cross-sectional design precludes causal inference; the Governance Gap is a concurrent asymmetry, not evidence that board AI literacy deficits cause executive AI overreach. The environmental sustainability dimension, while theoretically central, is not directly measured.

Priority directions for future research include: large-sample replication with stratified probability sampling; longitudinal designs tracking co-evolution of board AI literacy and environmental performance; development and validation of multi-item board AI governance capability instruments; behavioral measures supplementing self-report; and linkage studies connecting governance configurations to externally verified ESG disclosure quality.

## 7 Conclusion

This pilot study documents a measurable and statistically significant AI governance capability gap at the board level. Board AI literacy (M = 2.50) is significantly lower than executive AI competence (M = 3.70; *d* = 1.06, *p* = 0.026). AI auditing capacity (M = 1.79) and ethical AI oversight (M = 1.86) approach the measurement floor. Executive AI competence is tightly coupled with organizational AI vision orientation (*ρ* = 0.85, *p* = 0.002) without commensurate board scrutiny. Board members acknowledge the growing importance of AI governance (M = 3.21) while reporting near-zero operational capacity across every AI governance dimension. Taken as a whole, and within the evidentiary limits of a small exploratory pilot, the data are consistent with a governance tier that is formally responsible for AI oversight while reporting little of the technical capability that such oversight would require in the domains where it is most consequential. These results are signals to be tested at scale rather than settled findings.

The study’s primary practical contribution is the identification of board AI literacy as a missing governance capability in current corporate AI governance practice—a gap with direct implications for AI-driven ESG disclosure integrity and for the conditions under which ethical AI principles can be operationalized in sustainability governance. These findings are offered as preliminary empirical signals warranting a larger-scale investigation, not as definitive conclusions. If the patterns documented here replicate at scale, they would suggest that effective AI governance in corporate sustainability requires deliberative governance architecture grounded in demonstrable AI literacy at board level and independent AI auditing infrastructure, rather than structural governance elaboration alone. As such, the study should be read not as a definitive governance diagnosis, but as an early empirical warning that board-level AI capability may represent one of the least examined bottlenecks in the practical realization of ethical corporate AI governance.

### 7.1 Practical implications and preliminary governance recommendations

The following preliminary recommendations are offered on the basis of the pilot evidence presented, recognizing that the small, geographically concentrated sample limits the certainty with which these recommendations can be advanced. They should be understood as governance hypotheses—directions that the present findings suggest are worth investigating and piloting—rather than as evidence-based regulatory mandates. If the patterns documented here replicate in larger studies, the following governance directions would be indicated. None of the recommendations below should be read as supported by the present sample alone, which is too small and too narrowly drawn to sustain prescriptive guidance; they are framed as hypotheses for validation.

For boards of directors: Boards may benefit from distinguishing between AI awareness (recognizing the importance of AI governance) and AI auditing capacity (the operational ability to evaluate AI system outputs). Where this gap exists, boards might consider designating a specific AI oversight function with access to independent technical expertise, and requiring that AI-generated ESG data be accompanied by documentation of the systems, training data, and methodological assumptions that produced it prior to board endorsement.

For corporate governance regulators: If the Governance Gap documented here proves generalizable, governance quality assessment frameworks might benefit from distinguishing AI governance structure compliance (committee existence, policy adoption) from AI governance capability (demonstrated board-level ability to evaluate AI outputs in governance-relevant domains). Preliminary evidence of the gap between board AI literacy and AI auditing capacity suggests that structure-based assessment alone may be insufficient.

For institutional investors: Investors conducting ESG due diligence might consider whether board-level AI governance capability assessments—distinct from general digital literacy declarations—provide additional predictive value for sustainability disclosure reliability. The motivated non-preparation pattern documented here (awareness coexisting with near-zero operational capacity) may represent an observable governance quality indicator.

## Data Availability

The raw data supporting the conclusions of this article will be made available by the authors, without undue reservation.
